# Clinical and histopathological predictors of secondary arteriovenous fistula failure due to intimal hyperplasia

**DOI:** 10.3389/fphys.2026.1846551

**Published:** 2026-07-22

**Authors:** Fulong Wen, Zhengsheng Li, Juan Xie

**Affiliations:** Department of Nephrology, The Second Affiliated Hospital of Guizhou University of Traditional Chinese Medicine, Guiyang, China

**Keywords:** arteriovenous fistula, hemodialysis, intimal hyperplasia, nomogram, predictive model, vascular access failure

## Abstract

**Background:**

Intimal hyperplasia (IH) is an important pathological mechanism underlying secondary arteriovenous fistula (AVF) failure in previously mature fistulas, yet the clinical and histopathological determinants that distinguish progressive IH leading to access loss from stable IH remain poorly characterized. This study aimed to identify clinical and histopathological predictors of secondary AVF failure due to IH in previously mature fistulas, and to develop a predictive nomogram for risk stratification.

**Methods:**

This retrospective cohort study enrolled 320 patients with end-stage renal disease (ESRD) who underwent AVF creation between January 2020 and June 2025 and had histopathologically confirmed IH from surgical specimens. Patients were classified into a secondary failure group (n = 142, previously mature AVFs permanently abandoned due to IH-driven dysfunction) and a secondary patency group (n = 178) based on access outcomes. Clinical, laboratory, hemodynamic, and histopathological variables were compared between groups. Multivariable logistic regression was performed to identify independent predictors. A nomogram was constructed and internally validated using bootstrap resampling (1,000 iterations).

**Results:**

Multivariable analysis identified seven independent predictors of secondary AVF failure: diabetes mellitus (OR = 2.31, 95% CI: 1.42–3.76, P = 0.001), C-reactive protein ≥ 8.0 mg/L (OR = 1.89, 95% CI: 1.18–3.03, P = 0.008), intima-to-media thickness ratio > 1.5 (OR = 3.67, 95% CI: 2.18–6.17, P < 0.001), CD68^+^ macrophage density > 25 cells/HPF (OR = 2.54, 95% CI: 1.53–4.22, P < 0.001), α-SMA positive area > 40% (OR = 2.12, 95% CI: 1.31–3.43, P = 0.002), MMP-9 high expression (OR = 1.95, 95% CI: 1.21–3.14, P = 0.006), and neovascularization density > 15 vessels/HPF (OR = 1.78, 95% CI: 1.09–2.91, P = 0.021). The nomogram demonstrated good discrimination (C-index = 0.847, 95% CI: 0.803–0.891) and calibration. Decision curve analysis confirmed superior net clinical benefit compared with individual predictors.

**Conclusions:**

Integration of clinical parameters and histopathological markers enables accurate prediction of secondary AVF failure due to IH. The proposed nomogram may facilitate individualized risk stratification and guide clinical decision-making regarding vascular access management in hemodialysis patients.

## Introduction

Vascular access remains the Achilles’ heel of hemodialysis therapy for patients with end-stage renal disease (ESRD). Among the three principal forms of vascular access—arteriovenous fistula (AVF), arteriovenous graft (AVG), and central venous catheter (CVC)—the autogenous AVF is universally recommended as the preferred modality owing to its superior long-term patency, lower complication rates, and reduced healthcare costs (Lok et al., 2020; Schmidli et al., 2018). Despite these advantages, AVF failure continues to pose a substantial clinical burden, with reported primary failure rates of 20–60% and cumulative patency rates declining over time (Al-Jaishi et al., 2014; Huber et al., 2021).

Intimal hyperplasia (IH) is an important pathological mechanism underlying secondary AVF failure (loss of patency in previously mature fistulas) and late stenosis. While poor outward remodeling and venous fibrosis may predominate during initial maturation failure, IH-driven luminal compromise represents a major and clinically distinct cause of secondary access loss (Granata et al., 2025). The pathogenesis of IH involves a complex interplay of hemodynamic perturbations, endothelial dysfunction, smooth muscle cell (SMC) migration and proliferation, extracellular matrix remodeling, inflammatory cell infiltration, and neovascularization (Brahmbhatt et al., 2016; Rothuizen et al., 2013). Upon AVF creation, the venous endothelium is exposed to arterial hemodynamic forces, including elevated shear stress and cyclic strain, which trigger a cascade of molecular events culminating in neointimal formation (Browne et al., 2015).

However, not all AVFs with histological evidence of IH progress to clinical failure. A substantial proportion of functioning AVFs demonstrate varying degrees of intimal thickening that remains hemodynamically insignificant (Duque et al., 2018). This observation raises a critical question: what distinguishes “aggressive” IH that leads to access failure from “benign” IH that permits sustained AVF function? Identifying the determinants that differentiate these two trajectories has important implications for clinical practice, as it would enable risk stratification at the time of surgical intervention and inform decisions regarding surveillance intensity, preemptive treatment, and access planning (Robbin et al., 2002, 2018). The answer to this critical question may also inform improvements in endovascular treatments to prevent restenosis and secondary access loss.

Previous studies have predominantly focused on either clinical risk factors or histopathological features in isolation, with limited attempts to integrate both domains into a comprehensive predictive framework (Dixon et al., 2009; Wasse et al., 2012; Martinez et al., 2018, 2019). Clinical variables such as diabetes mellitus, advanced age, and systemic inflammation have been associated with AVF failure (Yan et al., 2018; Jeong et al., 2019; Kaygin et al., 2013), while histopathological markers including intimal thickness, inflammatory infiltration, and smooth muscle cell accumulation have been linked to IH severity (Allon et al., 2013; Lee et al., 2011b; Alpers et al., 2017). Nevertheless, the relative contributions of these factors and their combined predictive capacity remain insufficiently characterized.

Nomograms have emerged as valuable clinical tools for individualized risk prediction across various medical disciplines, offering intuitive graphical representations of multivariable regression models (Balachandran et al., 2015). To our knowledge, no previous study has developed a nomogram integrating clinical and histopathological variables specifically for predicting AVF failure attributable to IH.

Therefore, the present study aimed to: (1) compare clinical characteristics and histopathological features between patients with secondary AVF failure due to IH and those with patent AVFs despite histological IH; (2) identify independent predictors of secondary AVF failure through multivariable analysis; and (3) construct and validate a nomogram for individualized risk prediction of secondary AVF failure due to IH.

## Materials and methods

### Study design and population

This single-center retrospective cohort study was conducted at The Second Affiliated Hospital of Guizhou University of Traditional Chinese Medicine, China, between January 2020 and June 2025. The study protocol was approved by the Institutional Ethics Committee (approval number: 2025-274), and informed consent was obtained from all participants. The study was performed in accordance with the Declaration of Helsinki and reported following the STROBE guidelines for observational studies.

We reviewed the medical records of all ESRD patients who underwent autogenous AVF creation or revision surgery during the study period. Patients were eligible for inclusion if they met the following criteria: (1) age ≥ 18 years; (2) autogenous AVF (radiocephalic or brachiocephalic) created for hemodialysis; (3) availability of vascular tissue specimens obtained during AVF revision, thrombectomy, or abandonment surgery with histopathologically confirmed IH; (4) complete clinical and laboratory data; and (5) minimum follow-up of 6 months. The follow-up period was measured from the date of the index surgical procedure (revision, thrombectomy, or abandonment) at which tissue specimens were obtained. Follow-up continued through the end of the study observation period (June 2025) or until permanent access abandonment, whichever occurred first. The minimum 6-month follow-up requirement was applied to confirm sustained patency in the maintained-patency group and to avoid premature outcome classification; for patients who reached the secondary-failure endpoint, follow-up ended on the date of permanent abandonment. The proposed model is intended for application at the time of AVF revision or thrombectomy—when histopathological material first becomes available—to estimate the probability that a previously mature access will ultimately be lost despite salvage attempts versus retain function, thereby informing the intensity of salvage efforts and the timing of alternative-access planning.

Exclusion criteria were: (1) prosthetic arteriovenous graft; (2) AVF failure attributable to acute thrombosis without evidence of underlying IH; (3) infection-related access failure; (4) inadequate tissue specimens for histopathological analysis; and (5) incomplete medical records.

A total of 320 patients fulfilling the eligibility criteria were enrolled. In accordance with standard KDOQI/KDIGO vascular-access terminology, patients were classified by access outcome into a secondary failure group and a secondary patency group. Secondary failure (secondary failure group, n = 142) was defined as irreversible loss of patency of a previously mature, functional AVF requiring permanent abandonment or conversion to an alternative access modality owing to IH-induced stenosis (access blood flow < 400 mL/min despite intervention or complete thrombotic occlusion secondary to IH). Secondary patency (secondary patency group, n = 178) denoted patients whose AVFs demonstrated histological IH on specimens obtained during elective revision or biopsy but retained adequate function (sustained access blood flow ≥ 400 mL/min) throughout the follow-up period.

### Clinical data collection

Demographic and clinical data were extracted from electronic medical records, including age, sex, body mass index (BMI), comorbidities (diabetes mellitus, hypertension, coronary artery disease, peripheral arterial disease), smoking history, dialysis vintage, duration of AVF use, AVF location (forearm vs. upper arm), number of previous AVF surgeries, and medications (antiplatelet agents, statins, antihypertensives).

Laboratory parameters were recorded from the most recent blood sampling prior to the index surgical procedure, including serum albumin, C-reactive protein (CRP), hemoglobin, serum calcium, serum phosphorus, intact parathyroid hormone (iPTH), total cholesterol, triglycerides, low-density lipoprotein cholesterol (LDL-C), and glycated hemoglobin (HbA1c).

Hemodynamic parameters were assessed by duplex ultrasound within one month prior to surgery, including access blood flow rate (Qa), resistance index (RI), and luminal diameter at the stenotic segment. For accesses presenting with complete thrombotic occlusion at the index procedure, the luminal diameter at the culprit lesion was taken from the most recent duplex examination performed before the occlusive event; this value represents the residual (pre-occlusion) caliber at the stenotic segment rather than the post-occlusion lumen and was therefore non-zero.

### Tissue specimen collection and processing

Vascular tissue specimens were obtained during surgical procedures for AVF revision, thrombectomy, or abandonment. The juxta-anastomotic venous segment, which represents the most common site of IH-related stenosis, was preferentially sampled (Roy-Chaudhury et al., 2007; Granata et al., 2025). Of the 320 tissue specimens, 241 (75.3%) were obtained from the juxta-anastomotic segment and 79 (24.7%) from the peri-anastomotic outflow vein. Specimens were immediately fixed in 10% neutral buffered formalin for 24 hours, processed through graded ethanol dehydration, and embedded in paraffin. Serial sections (4 μm thickness) were cut perpendicular to the vessel axis for subsequent histological and immunohistochemical analyses.

### Histopathological analysis

All histopathological assessments were performed by two experienced pathologists blinded to clinical outcomes, with discrepancies resolved by consensus with a third senior pathologist.

### Morphometric analysis

Hematoxylin and eosin (H&E)-stained sections were used for morphometric measurements. Intimal thickness (IT) was measured as the distance from the luminal surface to the internal elastic lamina at four equidistant points around the vessel circumference, and the mean value was calculated. Medial thickness (MT) was similarly measured from the internal to the external elastic lamina. The intima-to-media thickness ratio (I/M ratio) was computed as IT/MT.

### Special staining

Masson’s trichrome staining was performed to evaluate collagen deposition, quantified as the percentage of blue-stained area relative to the total intimal area using ImageJ software (NIH, Bethesda, MD, USA). Elastic van Gieson (EVG) staining was used to assess elastic fiber integrity, scored semi-quantitatively on a 4-point scale: 0, intact elastic fibers; 1, mild fragmentation (< 25%); 2, moderate fragmentation (25–50%); 3, severe fragmentation (> 50%).

### Immunohistochemistry

Immunohistochemical staining was performed using the standard streptavidin-biotin-peroxidase method. The following primary antibodies were employed: anti-CD34 (clone QBEnd/10, 1:200, Dako) for endothelial cells and neovascularization; anti-CD68 (clone KP1, 1:100, Dako) for macrophages; anti-α-smooth muscle actin (α-SMA, clone 1A4, 1:400, Sigma-Aldrich) for smooth muscle cells and myofibroblasts; anti-matrix metalloproteinase-2 (MMP-2, 1:200, Abcam); anti-matrix metalloproteinase-9 (MMP-9, 1:200, Abcam); anti-transforming growth factor-β1 (TGF-β1, 1:100, Abcam); and anti-vascular endothelial growth factor (VEGF, 1:200, Abcam).

Neovascularization density was quantified by counting CD34-positive microvessels in five randomly selected high-power fields (HPF, ×400) within the neointima, expressed as vessels per HPF. CD68-positive macrophage density was determined by counting positively stained cells in five HPFs, expressed as cells per HPF. α-SMA positive area was quantified as the percentage of positively stained area within the neointima using ImageJ. MMP-2, MMP-9, TGF-β1, and VEGF expression levels were evaluated using a semi-quantitative immunoreactive score (IRS) based on staining intensity (0–3) multiplied by the percentage of positive cells (0–4), yielding scores of 0–12, categorized as low (0–4), moderate (5–8), or high (9–12) expression. Only high-power fields containing adequate, well-preserved neointimal tissue that essentially filled the entire field were used for quantification; fields with processing artefacts, tissue folds, or incomplete (< 100%) tissue coverage were excluded to avoid sampling and measurement bias (Remmele and Stegner, 1987; Peden et al., 2022).

### Statistical analysis

Statistical analyses were performed using R software (version 4.3.1, R Foundation for Statistical Computing, Vienna, Austria) and SPSS (version 26.0, IBM Corp., Armonk, NY, USA). Continuous variables were expressed as mean ± standard deviation (SD) for normally distributed data or median (interquartile range, IQR) for skewed distributions. Categorical variables were presented as frequencies and percentages. Normality was assessed using the Shapiro-Wilk test.

Univariate comparisons between the secondary failure and secondary patency groups were conducted using independent-samples t-test or Mann-Whitney U test for continuous variables and chi-square test or Fisher’s exact test for categorical variables.

Variables with P < 0.10 in univariate analysis were entered into a multivariable binary logistic regression model using forward stepwise selection (entry criterion: P < 0.05; removal criterion: P > 0.10). Multicollinearity was assessed using variance inflation factors (VIF), with VIF > 5 indicating significant collinearity. Odds ratios (ORs) with 95% confidence intervals (CIs) were reported.

A nomogram was constructed based on the final multivariable logistic regression model using the rms package in R. The predictive performance was evaluated by discrimination and calibration. Discrimination was assessed using the concordance index (C-index) and receiver operating characteristic (ROC) curve analysis. The area under the ROC curve (AUC) was calculated and compared between the nomogram and individual predictors using the DeLong test. Calibration was evaluated using calibration curves with the Hosmer-Lemeshow goodness-of-fit test. Internal validation was performed using bootstrap resampling with 1,000 iterations to obtain the bias-corrected C-index.

Decision curve analysis (DCA) was conducted to evaluate the clinical utility of the nomogram by quantifying the net benefit across a range of threshold probabilities (Vickers and Elkin, 2006).

Subgroup analyses were performed stratified by diabetes status (diabetic vs. non-diabetic) and AVF location (forearm vs. upper arm) to assess the consistency of predictive performance.

A two-sided P value < 0.05 was considered statistically significant for all analyses.

## Results

### Baseline clinical characteristics

Among 320 enrolled patients, 142 (44.4%) experienced secondary AVF failure due to IH and 178 (55.6%) maintained patent AVFs. The baseline clinical characteristics of the two groups are summarized in **Table 1**. The mean age of the study population was 58.3 ± 12.7 years, with 187 (58.4%) males. The median dialysis vintage was 36 (18–60) months, and the median AVF use duration was 24 (12–48) months.

**Table 1:** Baseline clinical characteristics of the study population.

Variable	Total (n=320)	Failure (n=142)	Patent (n=178)	P value
Demographics				
Age (years)	58.3 ± 12.7	61.2 ± 11.8	56.0 ± 13.0	0.001
Male sex	187 (58.4)	79 (55.6)	108 (60.7)	0.357
BMI (kg/m²)	23.8 ± 3.5	24.1 ± 3.7	23.6 ± 3.4	0.215
Comorbidities				
Diabetes mellitus	138 (43.1)	78 (54.9)	60 (33.7)	<0.001
Hypertension	264 (82.5)	121 (85.2)	143 (80.3)	0.247
Coronary artery disease	72 (22.5)	40 (28.2)	32 (18.0)	0.027
Peripheral arterial disease	45 (14.1)	24 (16.9)	21 (11.8)	0.186
Smoking history	98 (30.6)	48 (33.8)	50 (28.1)	0.268
Dialysis-related parameters				
Dialysis vintage (months)	36 (18–60)	38 (20–65)	34 (16–56)	0.087
AVF use duration (months)	24 (12–48)	26 (14–50)	22 (10–46)	0.112
Forearm AVF location	193 (60.3)	95 (66.9)	98 (55.1)	0.028
Previous AVF surgeries ≥ 2	68 (21.3)	38 (26.8)	30 (16.9)	0.028
Laboratory parameters				
Serum albumin (g/L)	36.5 ± 4.5	35.2 ± 4.8	37.6 ± 4.1	<0.001
CRP (mg/L)	7.2 (3.5–12.8)	9.8 (5.2–16.3)	5.4 (2.8–10.1)	<0.001
Hemoglobin (g/L)	102.3 ± 15.6	100.1 ± 16.2	104.1 ± 14.8	0.024
Serum calcium (mmol/L)	2.18 ± 0.24	2.16 ± 0.26	2.20 ± 0.22	0.152
Serum phosphorus (mmol/L)	1.72 ± 0.48	1.78 ± 0.52	1.67 ± 0.44	0.042
iPTH (pg/mL)	285 (156–445)	302 (168–478)	270 (148–420)	0.091
Total cholesterol (mmol/L)	4.52 ± 1.08	4.61 ± 1.15	4.45 ± 1.02	0.196
Triglycerides (mmol/L)	1.85 (1.22–2.68)	1.92 (1.28–2.82)	1.78 (1.18–2.55)	0.168
LDL-C (mmol/L)	2.68 ± 0.82	2.74 ± 0.88	2.63 ± 0.77	0.238
HbA1c (%)	6.7 ± 1.7	7.2 ± 1.8	6.4 ± 1.5	<0.001
Hemodynamic parameters				
Access blood flow (mL/min)	416 ± 178	285 ± 112	520 ± 165	<0.001
Resistance index	0.64 ± 0.12	0.72 ± 0.11	0.58 ± 0.09	<0.001
Luminal diameter (mm)	2.8 ± 1.1	2.1 ± 0.8	3.4 ± 1.0	<0.001
Medications, n (%)				
Antiplatelet agents	215 (67.2)	92 (64.8)	123 (69.1)	0.402
Statins	168 (52.5)	78 (54.9)	90 (50.6)	0.430
ACEI/ARB	142 (44.4)	65 (45.8)	77 (43.3)	0.650
CCB	198 (61.9)	90 (63.4)	108 (60.7)	0.612

Data are presented as mean ± SD, median (IQR), or n (%), as appropriate.

BMI, body mass index; AVF, arteriovenous fistula; IQR, interquartile range; CRP, C-reactive protein; iPTH, intact parathyroid hormone; LDL-C, low-density lipoprotein cholesterol; HbA1c, glycated hemoglobin; ACEI, angiotensin-converting enzyme inhibitor; ARB, angiotensin receptor blocker; CCB, calcium channel blocker.

Compared with the secondary patency group, patients in the secondary failure group were significantly older (61.2 ± 11.8 vs. 56.0 ± 13.0 years, P = 0.001), had a higher prevalence of diabetes mellitus (54.9% vs. 33.7%, P < 0.001), and were more likely to have a history of coronary artery disease (28.2% vs. 18.0%, P = 0.027). The secondary failure group also exhibited significantly higher CRP levels (9.8 [5.2–16.3] vs. 5.4 [2.8–10.1] mg/L, P < 0.001), lower serum albumin (35.2 ± 4.8 vs. 37.6 ± 4.1 g/L, P < 0.001), and higher HbA1c (7.2 ± 1.8% vs. 6.4 ± 1.5%, P < 0.001). The forearm AVF location was more prevalent in the secondary failure group (66.9% vs. 55.1%, P = 0.028). No significant differences were observed in sex, BMI, hypertension prevalence, smoking history, or lipid profile between groups (**Table 1**).

Hemodynamic assessment revealed that the secondary failure group had significantly lower pre-operative access blood flow (285 ± 112 vs. 520 ± 165 mL/min, P < 0.001), higher resistance index (0.72 ± 0.11 vs. 0.58 ± 0.09, P < 0.001), and smaller luminal diameter at the stenotic site (2.1 ± 0.8 vs. 3.4 ± 1.0 mm, P < 0.001).

### Histopathological characteristics

Histopathological parameters are presented in **Table 2**. The secondary failure group demonstrated significantly greater intimal thickness (1.42 ± 0.58 vs. 0.78 ± 0.35 mm, P < 0.001) and higher I/M ratio (2.35 ± 1.12 vs. 1.18 ± 0.54, P < 0.001) compared with the secondary patency group. Neovascularization density was markedly elevated in the secondary failure group (22.4 ± 9.6 vs. 11.3 ± 5.8 vessels/HPF, P < 0.001), as was CD68^+^ macrophage density (32.7 ± 14.5 vs. 16.8 ± 8.2 cells/HPF, P < 0.001).

**Table 2:** Histopathological Characteristics of the Study Population.

Variable	Failure (n=142)	Patent (n=178)	P value
Morphometric parameters			
Intimal thickness (mm)	1.42 ± 0.58	0.78 ± 0.35	<0.001
Medial thickness (mm)	0.68 ± 0.22	0.71 ± 0.20	0.218
I/M ratio	2.35 ± 1.12	1.18 ± 0.54	<0.001
Special staining			
Collagen deposition (%)	42.5 ± 13.6	28.7 ± 10.4	<0.001
Elastic fiber fragmentation score	2 (1–3)	1 (0–2)	<0.001
Immunohistochemistry			
CD34^+^ neovascularization density (vessels/HPF)	22.4 ± 9.6	11.3 ± 5.8	<0.001
CD68^+^ macrophage density (cells/HPF)	32.7 ± 14.5	16.8 ± 8.2	<0.001
α-SMA positive area (%)	48.3 ± 15.2	31.6 ± 11.8	<0.001
MMP-2 expression			<0.001
Low (IRS 0–4)	38 (26.8)	72 (40.4)	
Moderate (IRS 5–8)	50 (35.2)	72 (40.4)	
High (IRS 9–12)	54 (38.0)	34 (19.1)	
MMP-9 expression			<0.001
Low (IRS 0–4)	30 (21.1)	68 (38.2)	
Moderate (IRS 5–8)	47 (33.1)	72 (40.4)	
High (IRS 9–12)	65 (45.8)	38 (21.3)	
TGF-β1 expression			<0.001
Low (IRS 0–4)	32 (22.5)	65 (36.5)	
Moderate (IRS 5–8)	52 (36.6)	73 (41.0)	
High (IRS 9–12)	58 (40.8)	40 (22.5)	
VEGF expression			<0.001
Low (IRS 0–4)	28 (19.7)	58 (32.6)	
Moderate (IRS 5–8)	53 (37.3)	76 (42.7)	
High (IRS 9–12)	61 (43.0)	44 (24.7)	

Data are presented as mean ± SD, median (IQR), or n (%), as appropriate.

I/M ratio, intima-to-media thickness ratio; IQR, interquartile range; HPF, high-power field; α-SMA, α-smooth muscle actin; MMP, matrix metalloproteinase; TGF-β1, transforming growth factor-β1; VEGF, vascular endothelial growth factor; IRS, immunoreactive score.

The α-SMA positive area, reflecting smooth muscle cell and myofibroblast accumulation within the neointima was significantly higher in the secondary failure group (48.3 ± 15.2% vs. 31.6 ± 11.8%, P < 0.001). Collagen deposition assessed by Masson’s trichrome staining was greater in the secondary failure group (42.5 ± 13.6% vs. 28.7 ± 10.4%, P < 0.001). Elastic fiber fragmentation scores were significantly higher in the secondary failure group (median 2 [1–3] vs. 1 [0–2], P < 0.001).

**Figure 1:**
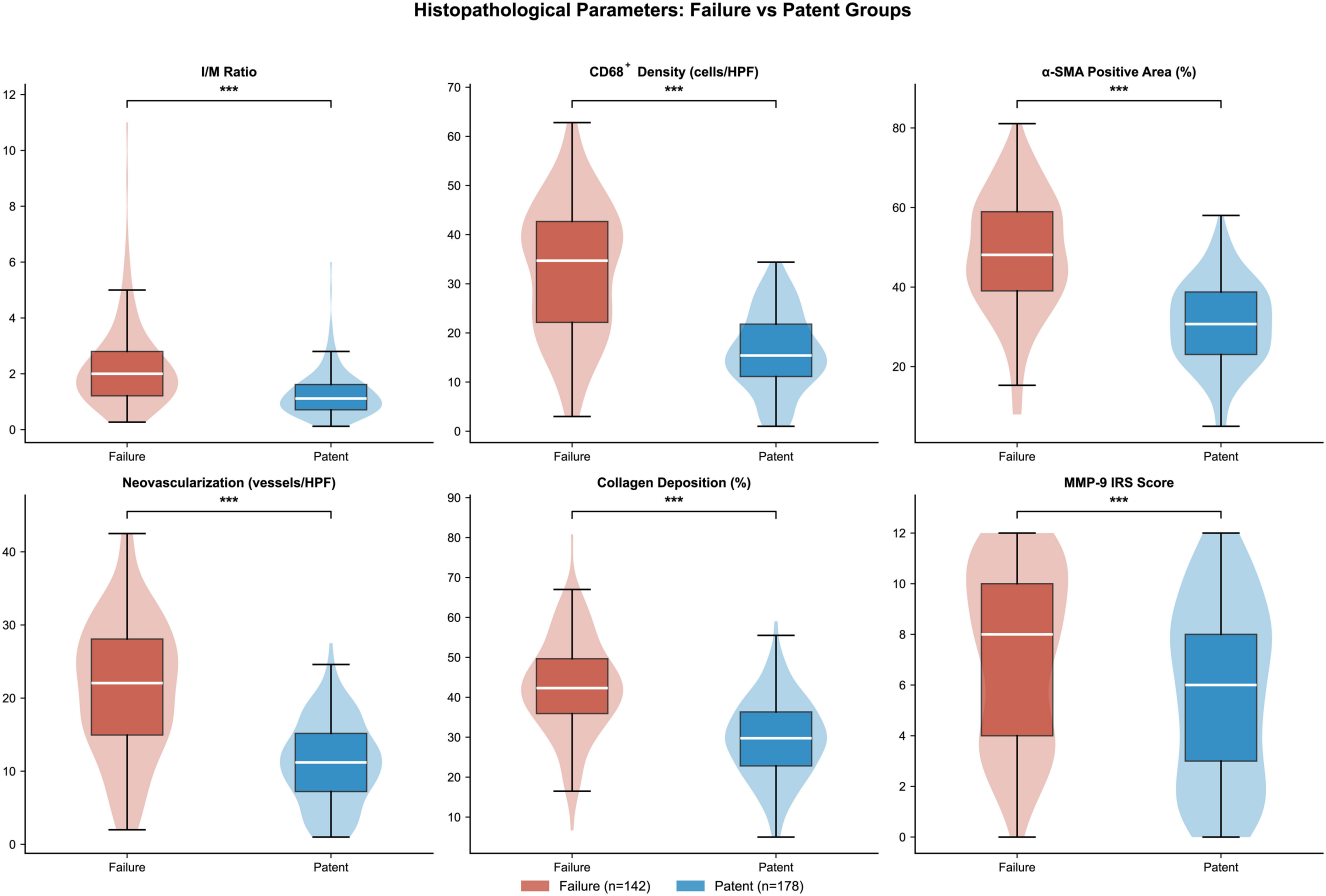
Comparison of histopathological parameters between failure and patent groups. Box-violin plots showing the distribution of key histopathological markers. ***P < 0.001.

**Figure 2:**
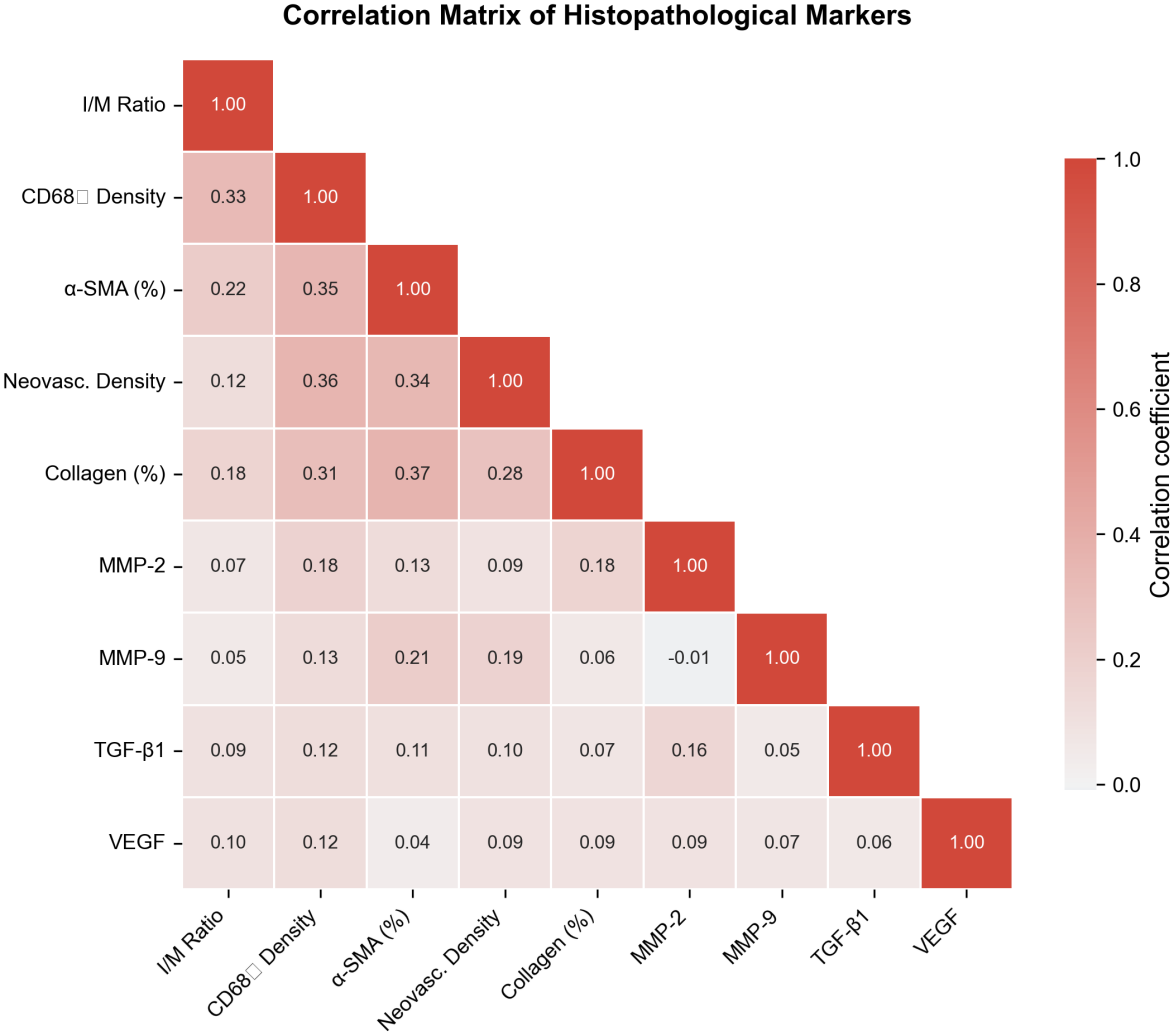
Correlation matrix of histopathological markers. Lower triangular heatmap showing Pearson correlation coefficients among histopathological variables.

Regarding molecular markers, the proportions of high expression (IRS 9–12) for MMP-2 (38.0% vs. 19.1%, P < 0.001), MMP-9 (45.8% vs. 21.3%, P < 0.001), TGF-β1 (40.8% vs. 22.5%, P < 0.001), and VEGF (43.0% vs. 24.7%, P < 0.001) were all significantly elevated in the secondary failure group compared with the secondary patency group (**Table 2**; **Figure 1**). Correlation analysis among histopathological markers revealed moderate-to-strong positive correlations between inflammatory markers (CD68^+^ density) and matrix remodeling indices (MMP-9, collagen deposition), supporting the concept of an interconnected pathological network driving IH progression (**Figure 2**).

### Multivariable logistic regression analysis

**Figure 3:**
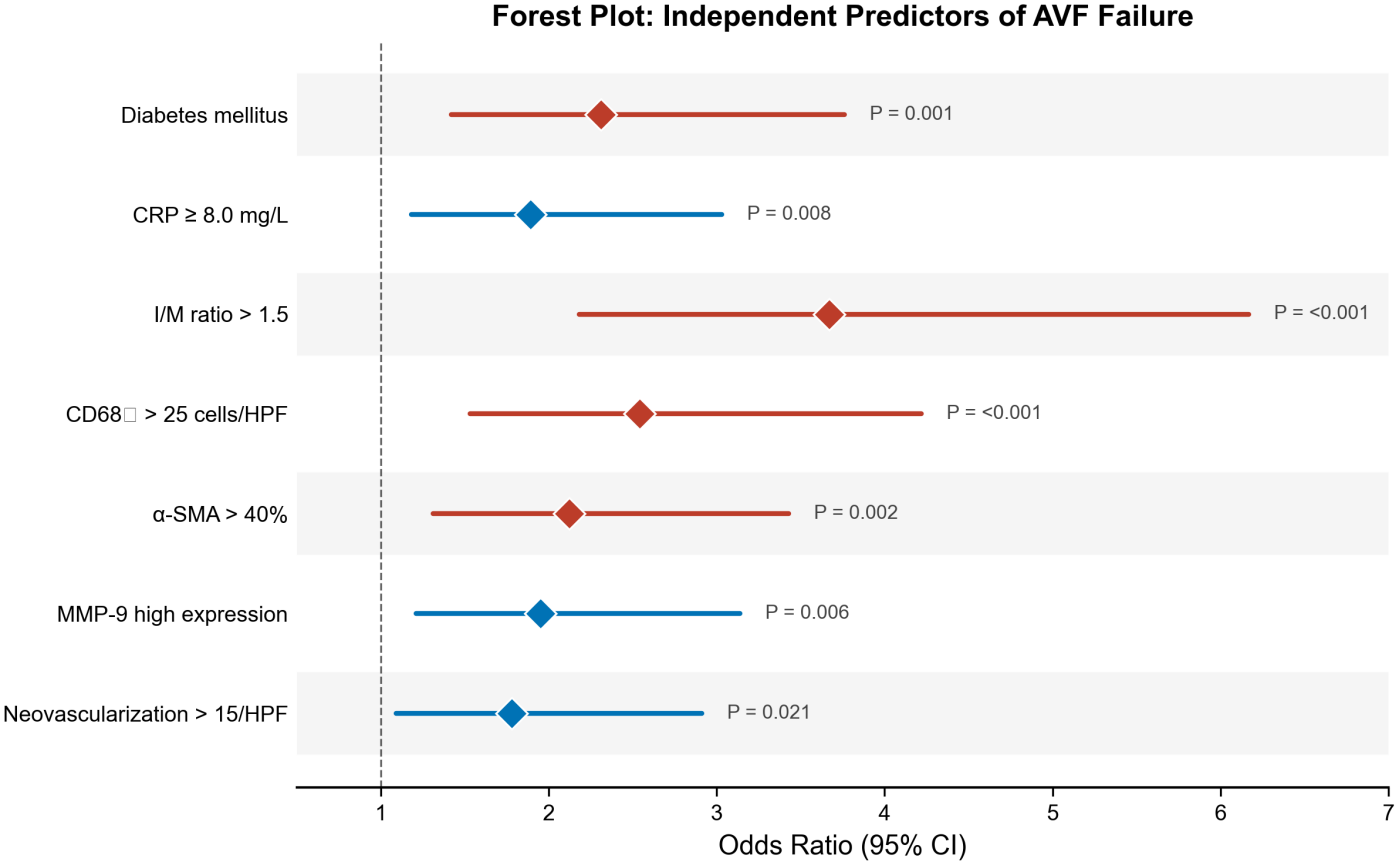
Forest plot of independent predictors of AVF failure from multivariable logistic regression. Diamond markers represent odds ratios with horizontal lines indicating 95% confidence intervals. The vertical dashed line at OR = 1 represents the null hypothesis.

Variables with P < 0.10 in univariate analysis were entered into the multivariable logistic regression model. Multicollinearity assessment confirmed that all VIF values were below 3.5, indicating acceptable collinearity levels. The final model identified seven independent predictors of AVF failure due to IH (**Table 3**; **Figure 3**): (1) Diabetes mellitus (OR = 2.31, 95% CI: 1.42–3.76, P = 0.001); (2) CRP ≥ 8.0 mg/L (OR = 1.89, 95% CI: 1.18–3.03, P = 0.008); (3) I/M ratio > 1.5 (OR = 3.67, 95% CI: 2.18–6.17, P < 0.001); (4) CD68^+^ macrophage density > 25 cells/HPF (OR = 2.54, 95% CI: 1.53–4.22, P < 0.001); (5) α-SMA positive area > 40% (OR = 2.12, 95% CI: 1.31–3.43, P = 0.002); (6) MMP-9 high expression (OR = 1.95, 95% CI: 1.21–3.14, P = 0.006); (7) Neovascularization density > 15 vessels/HPF (OR = 1.78, 95% CI: 1.09–2.91, P = 0.021).

**Table 3:** Multivariable logistic regression analysis of independent predictors of AVF failure due to intimal hyperplasia.

Variable	β	OR	95% CI	P value
Diabetes mellitus	0.838	2.31	1.42–3.76	0.001
CRP ≥ 8.0 mg/L	0.636	1.89	1.18–3.03	0.008
I/M ratio > 1.5	1.300	3.67	2.18–6.17	<0.001
CD68^+^ macrophage density > 25 cells/HPF	0.932	2.54	1.53–4.22	<0.001
α-SMA positive area > 40%	0.751	2.12	1.31–3.43	0.002
MMP-9 high expression (IRS 9–12)	0.668	1.95	1.21–3.14	0.006
Neovascularization density > 15 vessels/HPF	0.577	1.78	1.09–2.91	0.021

Model performance: Hosmer-Lemeshow test: χ² = 8.42, P = 0.394; Nagelkerke R² = 0.412; Classification accuracy = 78.4%.

β, regression coefficient; OR, odds ratio; CI, confidence interval; CRP, C-reactive protein; I/M ratio, intima-to-media thickness ratio; HPF, high-power field; α-SMA, α-smooth muscle actin; MMP-9, matrix metalloproteinase-9; IRS, immunoreactive score.

### Nomogram construction and validation

**Figure 4:**
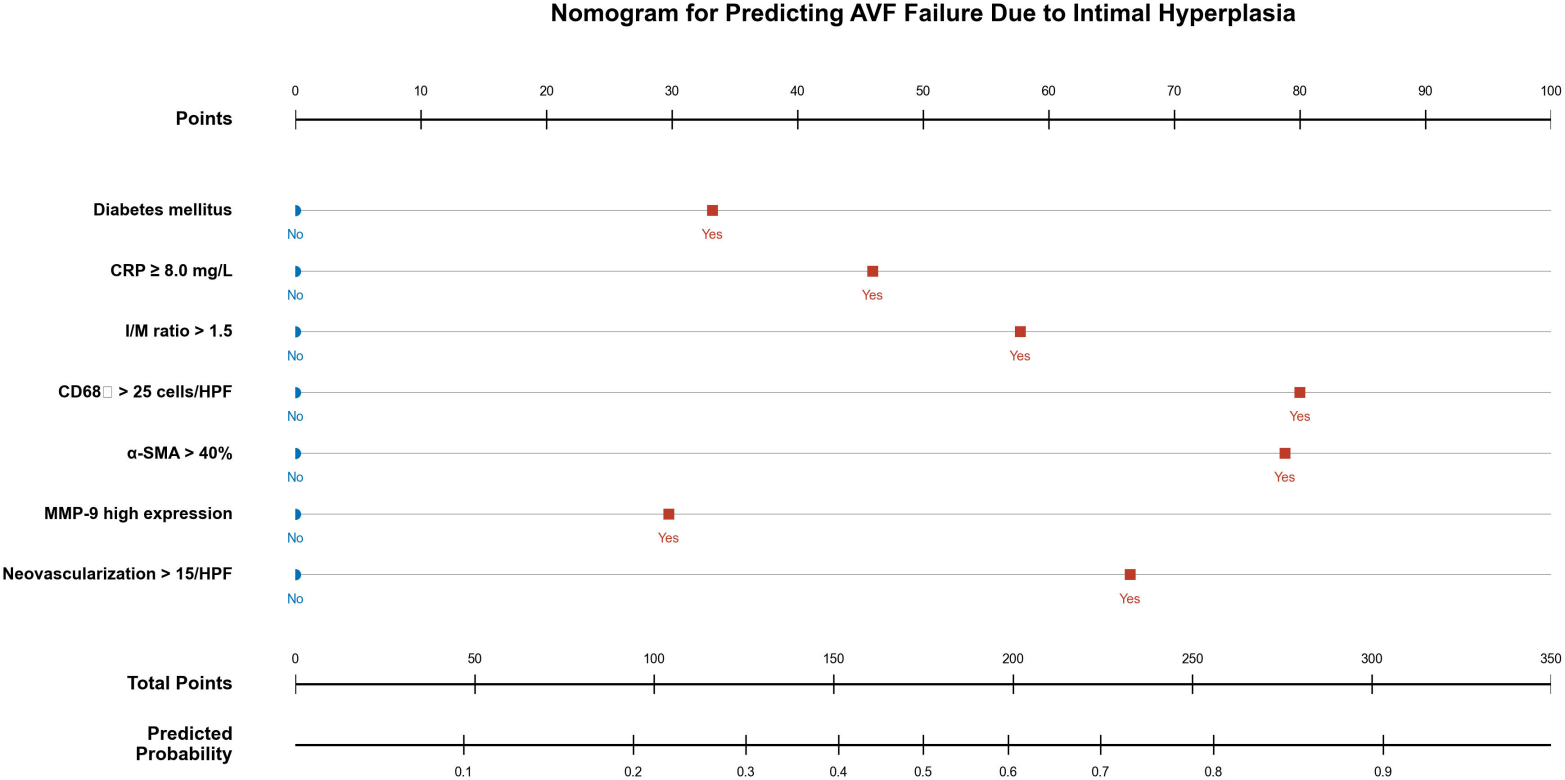
Nomogram for predicting AVF failure due to intimal hyperplasia. A nomogram incorporating seven independent predictors—diabetes mellitus, CRP ≥ 8.0 mg/L, intima-to-media thickness ratio > 1.5, CD68^+^ macrophage density > 25 cells/HPF, α-SMA positive area > 40%, MMP-9 high expression (IRS 9–12), and neovascularization density > 15 vessels/HPF—was constructed based on multivariable logistic regression analysis. Each predictor is assigned a point value on the top scale (Points). The total points, calculated by summing individual predictor points, correspond to the predicted probability of AVF failure on the bottom scale.

A nomogram incorporating the seven independent predictors was constructed to predict the probability of AVF failure due to IH (**Figure 4**). Each predictor was assigned a point score proportional to its regression coefficient, and the total points corresponded to the predicted probability of AVF failure.

**Figure 5:**
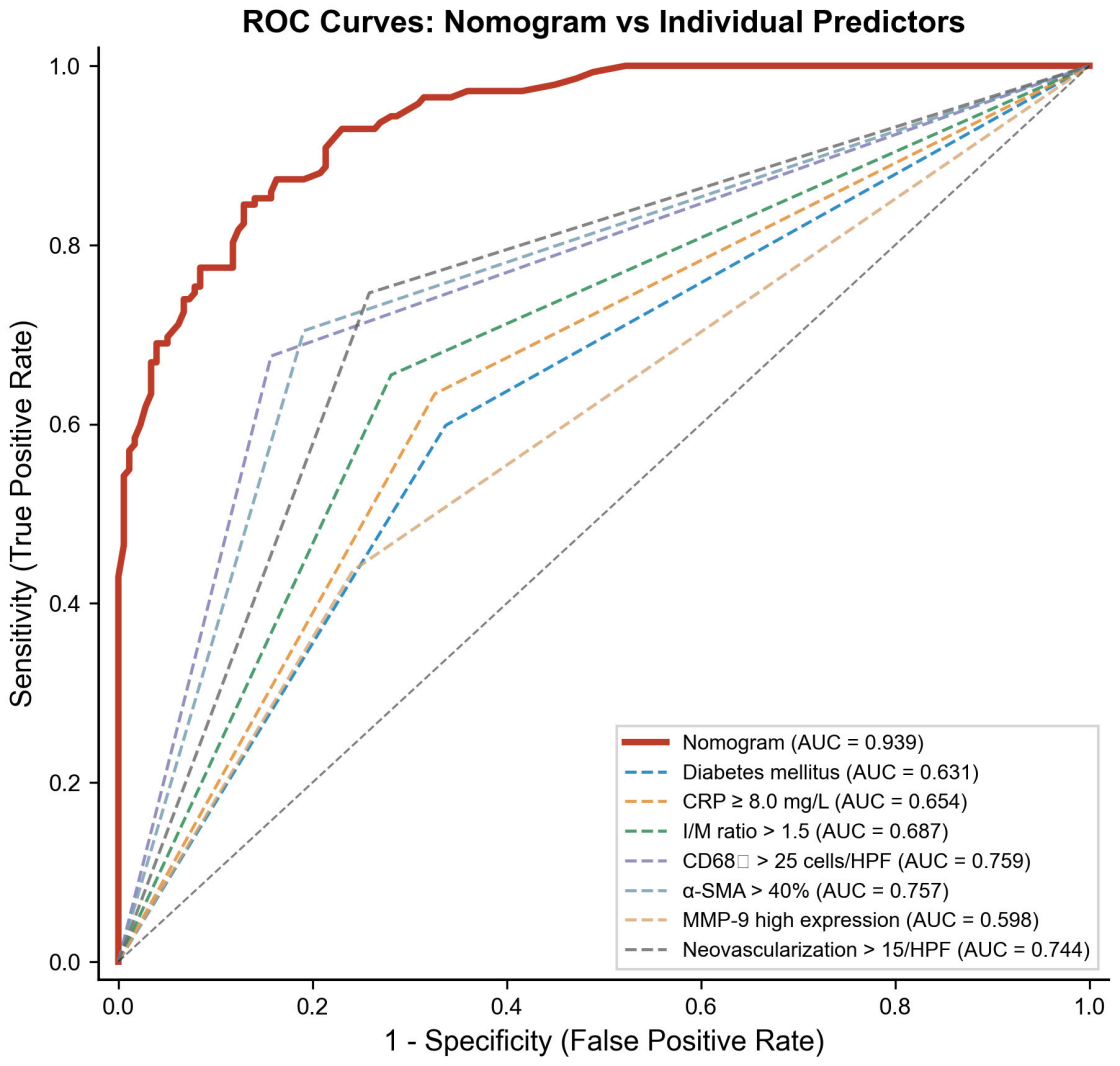
Receiver operating characteristic (ROC) curves comparing the discriminative performance of the nomogram and individual predictors. The nomogram (AUC = 0.847) demonstrated significantly superior discriminative ability compared with each individual predictor (all P < 0.01, DeLong test).

The nomogram demonstrated excellent discrimination, with a C-index of 0.847 (95% CI: 0.803–0.891). After bootstrap internal validation with 1,000 iterations, the bias-corrected C-index was 0.831, indicating minimal optimism. The ROC curve analysis yielded an AUC of 0.847, which was significantly higher than any individual predictor alone (all P < 0.01, DeLong test) (**Figure 5**).

**Figure 6:**
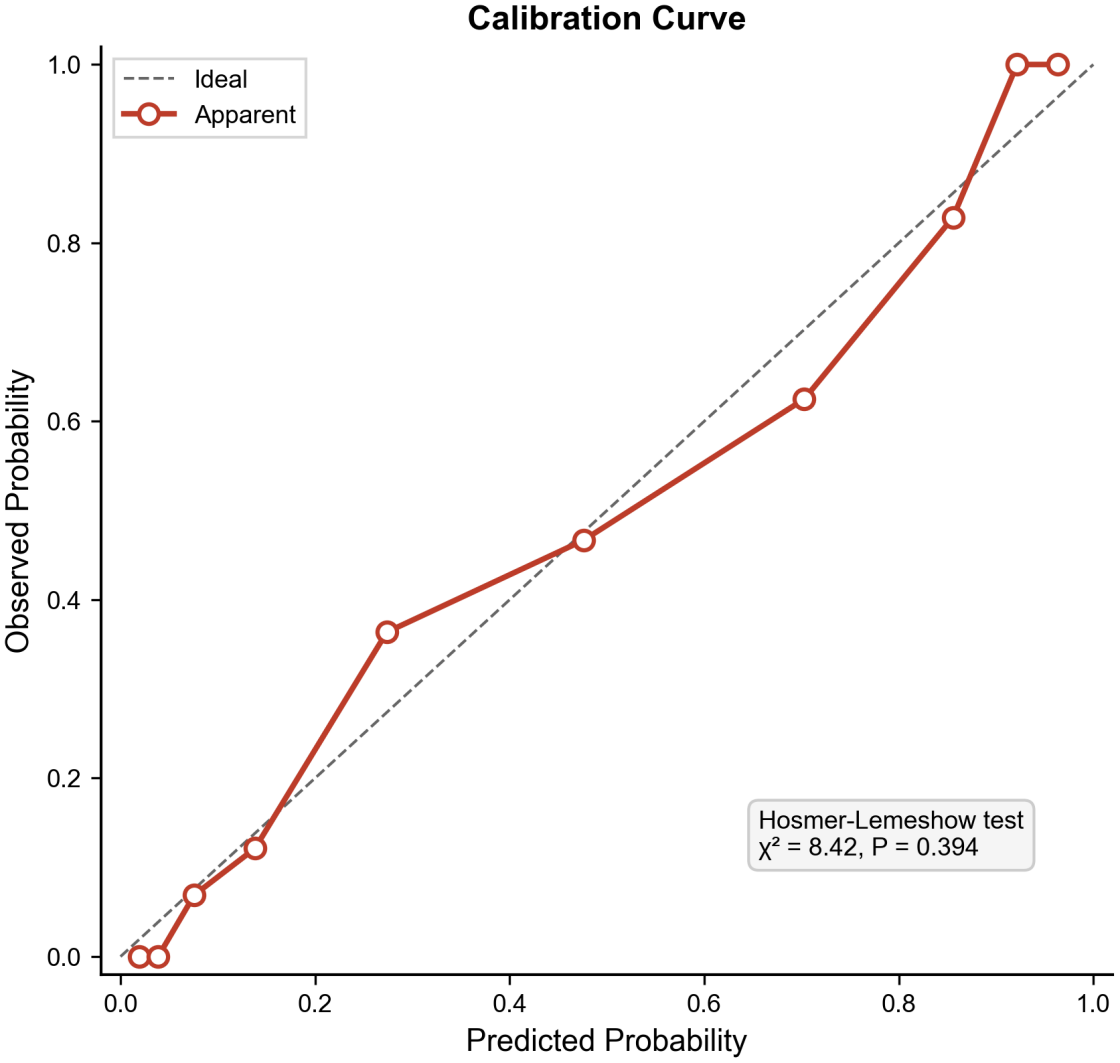
Calibration curve of the nomogram for predicting AVF failure due to intimal hyperplasia. The calibration curve demonstrates the agreement between the nomogram-predicted probability and the actual observed probability of AVF failure. The close approximation of the calibration curve to the ideal line indicates excellent calibration (Hosmer-Lemeshow test: χ² = 8.42, P = 0.394).

The calibration curve demonstrated good agreement between predicted and observed probabilities of AVF failure, with a non-significant Hosmer-Lemeshow test (χ² = 8.42, P = 0.394), indicating adequate calibration (**Figure 6**).

### Decision curve analysis

**Figure 7:**
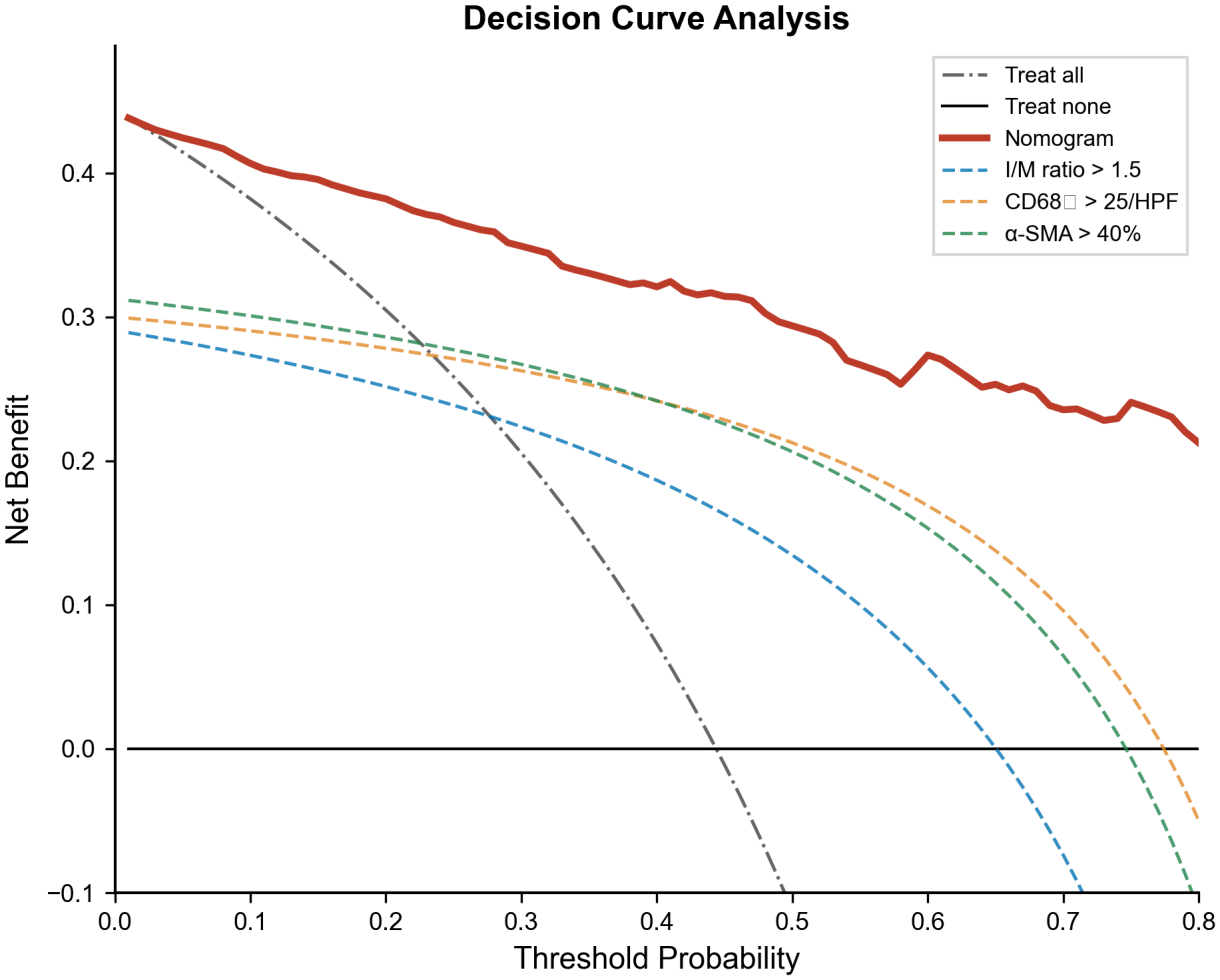
Decision curve analysis (DCA) of the nomogram for predicting AVF failure due to intimal hyperplasia. The nomogram provides greater net benefit than the treat-all strategy, treat-none strategy, and individual predictors across threshold probabilities of approximately 15–80%.

DCA revealed that the nomogram provided superior net benefit compared with individual predictors and treat-all or treat-none strategies across a wide range of threshold probabilities (approximately 15–80%), confirming its clinical utility for decision-making (**Figure 7**).

### Subgroup analysis

**Figure 8:**
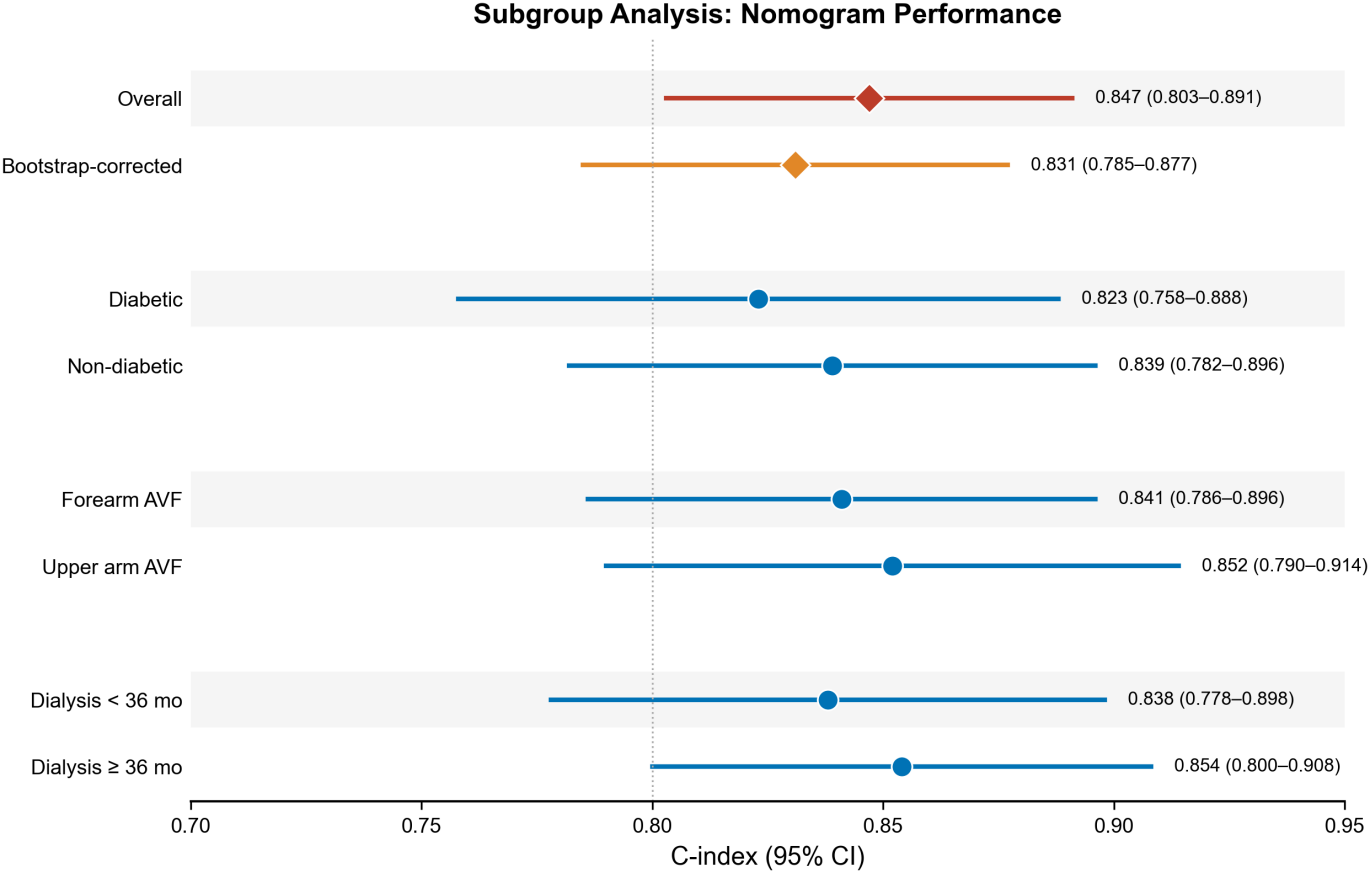
Subgroup analysis of nomogram predictive performance. Forest-style plot showing C-index with 95% confidence intervals for overall cohort, bootstrap-corrected, and stratified subgroups.

Stratified analysis by diabetes status demonstrated that the nomogram maintained good predictive performance in both diabetic (C-index = 0.823, 95% CI: 0.758–0.888) and non-diabetic (C-index = 0.839, 95% CI: 0.782–0.896) subgroups (**Table 4**; **Figure 8**). Similarly, the model performed consistently across forearm AVF (C-index = 0.841, 95% CI: 0.786–0.896) and upper arm AVF (C-index = 0.852, 95% CI: 0.790–0.914) subgroups. No significant interaction effects were observed between subgroup variables and the independent predictors (all P for interaction > 0.10).

**Table 4:** Subgroup analysis of nomogram predictive performance.

Subgroup	n	C-index (95% CI)	AUC (95% CI)
Overall cohort	320	0.847 (0.803–0.891)	0.847 (0.803–0.891)
Bootstrap-corrected	320	0.831 (0.785–0.877)	—
By diabetes status			
Diabetic	138	0.823 (0.758–0.888)	0.823 (0.758–0.888)
Non-diabetic	182	0.839 (0.782–0.896)	0.839 (0.782–0.896)
By AVF location			
Forearm	193	0.841 (0.786–0.896)	0.841 (0.786–0.896)
Upper arm	127	0.852 (0.790–0.914)	0.852 (0.790–0.914)
By dialysis vintage			
< 36 months	155	0.838 (0.778–0.898)	0.838 (0.778–0.898)
≥ 36 months	165	0.854 (0.800–0.908)	0.854 (0.800–0.908)

C-index, concordance index; AUC, area under the receiver operating characteristic curve; CI, confidence interval; AVF, arteriovenous fistula.

## Discussion

To our knowledge, this is the first study to systematically integrate clinical and intimal histopathological parameters to predict secondary AVF failure specifically attributable to IH. Our findings demonstrate that a combination of two clinical factors (diabetes mellitus and elevated CRP) and five intimal histopathological markers (I/M ratio, CD68^+^ macrophage density, α-SMA positive area, MMP-9 expression, and neovascularization density) independently predict AVF failure due to IH. The resultant nomogram exhibited excellent discriminative ability (C-index = 0.847) and calibration, offering a practical tool for individualized risk stratification.

The identification of diabetes mellitus as an independent predictor of secondary AVF failure aligns with extensive prior evidence (Yan et al., 2018; Jeong et al., 2019). Diabetes has been associated with IH through multiple proposed mechanisms, including advanced glycation end-product (AGE) accumulation, oxidative stress-induced endothelial dysfunction, and chronic low-grade inflammation (Goldin et al., 2006; Granata et al., 2025). In our cohort, diabetic patients exhibited significantly thicker intimal layers and greater inflammatory infiltration, consistent with a permissive role for dysglycemia in promoting aggressive IH in the AVF setting. Indeed, multiple clinical factors including diabetes, vessel characteristics, and patient demographics have been comprehensively analyzed as key determinants of AVF maturation outcomes, further corroborating the multifactorial nature of access failure (Siddiqui et al., 2017).

Elevated CRP, reflecting systemic inflammation, was independently associated with AVF failure, consistent with the growing recognition of inflammation as a key driver of vascular access dysfunction (Kaygin et al., 2013). Systemic inflammation may exacerbate local vascular pathology through upregulation of adhesion molecules, recruitment of inflammatory cells to the vessel wall, and enhancement of pro-fibrotic signaling cascades. The synergistic relationship between systemic inflammation and local histopathological changes underscores the importance of assessing both clinical and tissue-level parameters in predicting access outcomes.

Among histopathological predictors, the I/M ratio emerged as the strongest independent predictor (OR = 3.67), underscoring the primacy of structural vascular remodeling in determining AVF fate. An I/M ratio exceeding 1.5 indicates that intimal thickening has surpassed the medial layer, reflecting a degree of neointimal expansion that may compromise luminal patency, particularly when outward remodeling capacity is insufficient. This finding is consistent with morphometric observations that absolute intimal thickness alone does not reliably distinguish stenotic from non-stenotic AVF segments, underscoring the additional discriminative value of the intima-to-media ratio (Duque et al., 2018) by establishing a clinically meaningful threshold with independent predictive value. Experimental evidence from rodent AV fistula models has demonstrated that arterialized veins undergo progressive functional and structural remodeling, including intimal thickening and medial adaptation, further supporting the pathological relevance of the I/M ratio as a quantitative indicator of adverse vascular remodeling. Importantly, intimal wall thickening is not inherently maladaptive; when accompanied by sufficient outward remodeling it need not impair luminal diameter, as demonstrated by postoperative imaging analyses in the HFM trial (Li et al., 2022). In our cohort, the association of elevated I/M ratio with secondary failure likely reflects cases in which intimal expansion exceeded outward remodeling capacity (Langer et al., 2009). A markedly elevated I/M ratio may additionally reflect increased intimal stiffness and reduced compressibility, which could contribute to the resistance to endovascular correction observed in such lesions.

The significant contribution of CD68^+^ macrophage density to the predictive model highlights the central role of inflammation in IH pathogenesis. Macrophages orchestrate the fibroproliferative response through secretion of cytokines (IL-1β, IL-6, TNF-α), growth factors (PDGF, TGF-β), and matrix metalloproteinases that collectively promote SMC migration, proliferation, and extracellular matrix remodeling (Juncos et al., 2011; Sterpetti et al., 1993). The threshold of 25 cells/HPF identified in our analysis may represent a tipping point beyond which the inflammatory milieu overwhelms endogenous regulatory mechanisms, driving IH toward clinically significant stenosis.

The α-SMA positive area, reflecting SMC and myofibroblast accumulation (primarily via migration from the media and phenotypic transition), was independently predictive of secondary AVF failure. In the context of IH, medial SMCs undergo a transition from a contractile to a synthetic phenotype, characterized by enhanced matrix synthesis, migratory behavior, and altered cytoskeletal architecture (Martinez et al., 2019; Owens et al., 2004). An α-SMA positive area exceeding 40% in the neointima suggests a critical mass of accumulated SMCs and myofibroblasts sustaining progressive luminal narrowing. Importantly, recent evidence has emphasized that IH-mediated AVF failure cannot be fully explained by vessel size differences alone, and that qualitative histopathological features—including cellular composition, SMC phenotypic changes, and extracellular matrix remodeling patterns—play equally critical roles in determining access outcomes (Robbin et al., 2018; Vazquez-Padron et al., 2021; Duque et al., 2018).

MMP-9 high expression was identified as an independent predictor, consistent with its established role in extracellular matrix degradation and vascular remodeling (Newby, 2006). MMP-9 facilitates SMC migration through the internal elastic lamina by degrading basement membrane components and promotes neointimal expansion through matrix turnover (Newby, 2006; Lee et al., 2011a). Notably, MMP-9 was a stronger predictor than MMP-2 in our analysis, which may relate to the more pronounced association of MMP-9 with inflammatory-driven matrix remodeling, as MMP-9 is primarily derived from infiltrating macrophages and neutrophils. These findings are in line with emerging insights into the diverse molecular targets underlying dialysis vascular access failure, which have identified MMP-9 and other signaling molecules as potential therapeutic targets for preventing or attenuating IH progression in both AVF and AVG settings (Lee and Misra, 2016).

Neovascularization density, reflecting intraplaque angiogenesis, emerged as a significant but relatively modest predictor (OR = 1.78). Intraneointimal neovascularization contributes to IH progression by providing conduits for inflammatory cell infiltration and growth factor delivery, creating a self-sustaining cycle of inflammation and proliferation. The VEGF–neovascularization axis has been implicated in both atherosclerotic plaque vulnerability and IH progression, and our findings extend this association to the specific context of AVF failure.

The nomogram developed in this study offers several practical advantages. First, it integrates readily obtainable clinical variables with histopathological parameters that can be assessed from surgical specimens, making it applicable at the time of AVF revision or thrombectomy. Second, the graphical format facilitates intuitive interpretation and communication of risk to patients and clinicians. Third, the excellent C-index of 0.847, maintained after bootstrap validation (0.831), indicates robust discriminative capacity suitable for clinical application.

This study has several limitations that warrant acknowledgment. First, the retrospective single-center design introduces potential selection bias and limits generalizability. Multicenter prospective studies are needed to externally validate the nomogram. Second, histopathological specimens were obtained at the time of surgical intervention rather than at AVF creation, precluding assessment of baseline vascular histology and its temporal evolution. Third, the study population was exclusively Chinese, and ethnic differences in vascular biology and IH susceptibility may limit applicability to other populations. Moreover, emerging evidence indicates that sex-dependent differences in TGF-β1/BMP7 signaling and venous fibrosis may contribute to differential IH susceptibility between males and females, a factor not specifically addressed in our model and warranting consideration in future studies (Cai et al., 2020). Fourth, although internal validation was performed using bootstrap resampling, external validation in an independent cohort is essential before clinical implementation. Fifth, molecular markers were assessed by immunohistochemistry, which provides semi-quantitative data; future studies employing quantitative techniques such as Western blotting or qRT-PCR could enhance precision. Sixth, our histopathological analysis was confined to the intimal layer; the media and adventitia — including adventitial fibrosis and perivascular inflammation — were not systematically quantified, despite their potential roles in wall stiffness and outward remodeling capacity. Seventh, we did not analyze the correlation between histological I/M ratio and ultrasound-measured luminal diameter, which would have clarified the hemodynamic consequence of intimal expansion in the context of varying outward remodeling. Furthermore, tissue sampling was predominantly from the juxta-anastomotic segment (241/320, 75.3%), and our findings may therefore not fully capture IH burden in more distal outflow segments, whose involvement has been reported to vary along the AVF. Finally, the cohort was analysed as a single composite secondary-failure endpoint: the terminal failure morphology (refractory non-occlusive stenosis versus complete thrombotic occlusion) and the use of balloon-assisted maturation or early endovascular intervention during the initial maturation period were not systematically recorded in this retrospective dataset, precluding a formal subtype comparison. Whether the predictor profile differs by failure mode or by prior maturation-assisting intervention warrants dedicated evaluation in future prospective studies.

This study identifies seven independent predictors of secondary AVF failure due to IH, encompassing both clinical parameters (diabetes mellitus and elevated CRP) and histopathological markers (I/M ratio > 1.5, CD68^+^ macrophage density > 25 cells/HPF, α-SMA positive area > 40%, MMP-9 high expression, and neovascularization density > 15 vessels/HPF). The integrated nomogram demonstrates excellent predictive performance and may serve as a practical tool for individualized risk stratification in hemodialysis patients with previously mature AVFs undergoing surgical intervention. Future prospective multicenter studies are warranted to externally validate this model and evaluate its impact on clinical decision-making and patient outcomes.

## Data Availability

The original contributions presented in the study are included in the article/supplementary material. Further inquiries can be directed to the corresponding author.
